# Estrogen-functionalized liposomes grafted with glutathione-responsive sheddable chotooligosaccharides for the therapy of osteosarcoma

**DOI:** 10.1080/10717544.2018.1458920

**Published:** 2018-04-12

**Authors:** Xuelei Yin, Shuaishuai Feng, Yingying Chi, Jinhu Liu, Kaoxiang Sun, Chuanyou Guo, Zimei Wu

**Affiliations:** a School of Pharmacy, Key Laboratory of Molecular Pharmacology and Drug Evaluation, (Yantai University), Ministry of Education, Collaborative Innovation Center of Advanced Drug Delivery System and Biotech Drugs in Universities of Shandong, Yantai University, Yantai, China;; b Department of Orthopedic Surgery, Qingdao Municipal Hospital, Qingdao, China;; c School of Pharmacy, University of Auckland, Auckland, New Zealand

**Keywords:** Estrogen-functionalized, chotooligosaccharides, reduction-sensitivity, targeting, cationic liposomes, osteosarcoma

## Abstract

An estrogen (ES)-functionalized cationic liposomal system was developed and exploited for targeted delivery to osteosarcoma. Natural biocompatible chotooligosaccharides (COS, MW2-5 KDa) were covalently tethered to the liposomal surface through a disulfate bond (-SS-) to confer reduction-responsive COS detachment, whereas estrogen was grafted via polyethylene glycol (PEG 2 K) chain to achieve estrogen receptor-targeting. The liposomal carriers were prepared by the ethanol injection method and fluorescent anticancer drug doxorubicin (DOX) was loaded with ammonium sulfate gradient. The physicochemical properties, reduction-sensitivity, and the roles of estrogen on cellular uptake and tumor-targeting were studied. The Chol-SS-COS/ES/DOX liposomes were spherical with an average size about 110 nm, and high encapsulation efficiency. The liposomes were stable in physiological condition but rapidly release the payload in response to tumoral intracellular glutathione (20 mM). MTT cytotoxicity assay confirmed that Chol-SS-COS/ES/DOX liposomes exhibited higher cytotoxicity to MG63 osteosarcoma cells than to liver cells (LO2). Flow cytometry (FCM) and confocal laser scanning microscopy revealed that cellular uptake of Chol-SS-COS/ES/DOX liposomes by MG63, than the free DOX or Chol-SS-COS/DOX. *Ex vivo* fluorescence distribution study showed that the multifunctional liposomes selectively accumulated in the MG63 xenografts versus the organs. Chol-SS-COS/ES/DOX liposomes strongly inhibited the tumor growth and enhanced the animal survival rate. Overall, the COS grafted estrogen-functionalized cationic liposomes, fortified with glutathione-responsiveness, showed great potential for specific intracellular drug delivery to estrogen receptor-expressing tumors such as osteosarcoma.

## 1. Introduction

Liposomes are the most successful drug delivery nano-carriers for tumor targeted drug delivery (Rajan et al., 2017) through the enhanced permeability and retention (EPR) effect (Maeda, 2001). To improve the degree of internalization to cancer cells, specific ligand-anchored liposomes have been explored which can be specifically recognized by cancer cells through their over-expressed receptors, for example, the estrogen receptors (Wicki et al., 2015). To reach the target, long circulation of the liposomes in blood stream is favorable property (Gelperina et al., 2005). For this, hydrophilic polymers, typically polyethylene glycols (PEG) (Sultana et al., 2013), have been used to modify the nanocarriers. However, PEGylation is expensive and could also reduce cellular uptake by cancer cells (known as PEG dilemma). Chitosan derivatives (glycol chitosan) has been used to overcome this shortcoming by taking advantage of the cationic property of chitosan which enhance the interaction with negatively changed cell membrane (Yan et al., 2015).

Chitosan, a class of nontoxic linear polymers composed of N-acetyl-D-glucosamine and deacetylated glucosamine, has been used in drug delivery due to its excellent characteristics, such as biocompatibility, biodegradation, mucoadhesive and low-immunogenicity (Felt et al., 1998; Kumar et al., 2004), as well as antimicrobial (Zheng & Zhu, 2003), anti-inflammatory (Yoon et al., 2007), and antitumoral (Qin et al., 2006) activities, due to the presence of reactive functional groups, namely, hydroxyl and amino acid groups (Swiatkiewicz et al., 2015). Because chitosan with high molecular weight are insoluble in aqueous medium at pH above 6.3 (Okuyama et al., 2000), its hydrolysis products with a low degree of polymerization (2-20 D-glucosamine units), chitooligosaccharides (COS) have been explored as alternatives (Qin et al., 2006; Lodhi et al., 2014). Being polycationic, COS have mainly been employed for ocular drug delivery by taking advantage of excellent mucoadhesiveness (Luo et al., 2011). In our recent study using preclinical animal models, Yin et al. (2017) demonstrated that conjugation of liposomes with COS enhance tumor cell uptake and improved antitumor effect along with great survival rate via preferential accumulation of the encapsulated drug within the tumor. Therapeutic effect was further improved by anchoring COS on the cholesterol (Chol) in the liposomal bilayers via disulfide bond (-SS-) (Chol-SS-COS liposomes) by promoting cytoplasmic drug delivery in cancer cells. Disulfide bonds are stable under physiological environment of normal cells and tissues, but cleavages (Lu & Chang, 2010; Jhaveri et al., 2014) in the presence of high concentration of bioreducing agent glutathione (GSH) in the cytosol and subcellular compartments (2–10 mM) of many cancer cells (Schafer & Buettner, 2001; Li et al., 2012).

To improve the targeting efficiency, in this paper, Chol-SS-COS liposomes further equipped with a ligand, estrone (an endogenous estrogen, ES) via PEG2000 chains (Chol-SS-COS/ES) were developed as potential drug carriers to osteosarcoma. The functional polymer DSPE-PEG_2000_-ES was synthesized and used for fabricate liposomes so that -PEG_2000_-ES was sandwiched in the COS chains on liposomal surface. Estrone would enable the liposomes be specifically endocytosed through receptor-mediated pathway by osteosarcoma cells. After entering cells, the liposomes destabilized due to the cleavage of -SS- in response to the intracellular GSH, rapidly releasing the drug payload in the cytoplasm. Proof of concept was tested on *in vitro* and *in vivo* osteosarcoma models with doxorubicin (DOX) being used as a model drug. To highlight the functions of estrogen in targeting osteosarcoma, Chol-SS-COS liposomes were used as reference formulation in this study. Osteosarcoma is the most common malignant bone tumors in children and adolescents worldwide with high risks for early metastasis and high mortality (Mirabello et al., 2009; Allison et al., 2012; Bousquet et al., 2016). The existence of estrogen receptors in osteosarcoma has been reported (Svoboda et al., 2010). Furthermore, estrogen could inhibit etoposide-induced apoptosis of human osteosarcoma cells via mediating estrogen-β receptor (Kallio et al., 2008) therefore targeting estrogen receptor positive cells is clinically important.

## 2. Materials and methods

### 2.1. Materials

Chitooligosaccharides (MW2-5 kDa and degree of deacetylation of 75%) were purchased by Fengan Bio-Pharmaceutica Co., *Ltd*. (Zhejiang, China) and DSPE-PEG_2000_-COOH from Avanti Polar Lipids (Alabaster, AL). Doxorubicin hydrochloride (DOX•HCl, purity 99%), estrone, cholesterol (Chol), glutathione (GSH), 3,3′-dithiodipropionic acid (DTOP), succinic anhydride (SA), 1-ethyl-3-(3-dimethylaminopropyl) carbodiimide hydrochloride (EDC•HCl), N-hydroxysuccinimide (NHS), 4-dimethylaminopyridine (DMAP) were all obtained from Aladdin (Shanghai, China). N,N′-dicyclohexylcarbodiimide (DCC) was purchased from Sigma-Aldrich (USA). For cell culture studies, MG-63 cells were purchased from BeNa Cell Nanosoft Biotechnology LLC Collection (Beijing, China), and LO2 cells were gifted from Medicine and Pharmacy Research Center, Binzhou Medical University. The chemical agents 3-(4, 5-dimethylthiazol-2-yl)-2,5-diphenyl tetrazolium bromide (MTT) and Hoechst 33342 were obtained from Sigma-Aldrich (USA). Dulbecco’s modified Eagle medium (DMEM), and Roswell Park Memorial Institute (RPMI)-1640 medium were purchased from Thermo Fisher Scientific (Shanghai, China). Fetal bovine serum (FBS) was purchased from Shanghai Biotechnology Co., Ltd. All other chemicals were of analytical reagent grade and used without further purification.

### 2.2. Synthesis of DSPE-PEG_2000_-ES

The DSPE-PEG_2000_-ES was synthesized via an EDC coupling reaction (Paliwal et al., 2012). DSPE-PEG_2000_-COOH (0.57 g), EDC (0.056 g) and DMAP (0.033 g) was dissolved in DMSO (10 ml) solution with constant stirring for 3 h. Estrone (0.06 g) was dissolved in DMSO (2 ml) and added to the above solution dropwise, and the mixture was kept at room temperature for 3 days under stirring. The mixture was then dialyzed against water for 2 days and frozen for preservation. Yield was 63.2%. ^1^H NMR (400 MHz, DMSO-*d*_6_) *δ* 0.82, 0.83 (ppm, CH_3_ of estrone and DSPE), 7.06, 6.52, 6.44 (ppm, CH of benzyl), 5.57 (d, J = 8.0 Hz, 5 H), 3.53 (ppm, –CH_2_–CH_2_– of mPEG_2000_).

### 2.3. Preparation of DOX-loaded liposomes

The Chol-SS-COS/ES liposomes (Chol-SS-COS/ES-Lp) and references, Chol-COS liposomes (Chol-COS-Lp) (non GHS sensitive) and Chol-SS-COS liposomes (Chol-SS-COS-Lp, with no estrone) were prepared by ethanol injection method as described previously (Yin et al., 2017). Briefly, SPC, Chol-SS-COOH or Chol-COOH, with or without DSPE-PEG_2000_-ES (weight ratio 2: 0.7: 0.3 or 0, total lipids about 30 mg for each batch) were added in 1 ml ethanol and sonicated for 10 min. The lipid solution was slowly added into 10 ml PBS (pH 7.4) dropwise with stirring 1 h to get the liposomes colloidal suspension. Immediately, COS (30 mg), along with EDC (4.6 mg) and NHS (2.7 mg) were added to the above resultant liposomes, and to allow COS (containing –NH_2_) tethered on the liposomes surface through formation of amide bond.

DOX was loaded into the liposomes using the ammonium sulfate gradient method as described previously (Yin et al., 2017). The free DOX was separated from the DOX-loaded liposomes by dialysis (MWCO 10 KDa) before further use.

### 2.4. Characterization of polymers and liposomes

The structure of polymers compound was characterized by Fourier transform infrared spectrometer (FT-IR, PerkinElmer Frontier, UK) and nuclear magnetic resonance spectrometer (^1^H NMR, Bruker ARX-400, Switzerland).

The particle size and zeta-potential of DOX-loaded liposomes were measured by dynamic light scattering (DLS, Malvern Instruments *Ltd*., UK) and morphology observed with a JEOL transmission electron microscope (TEM, JEM-1400, Japan).

To determine the amount of DOX loaded into liposomes DOX-loaded liposomes were freeze-dried and dissolved in ethanol. DOX concentrations in the supernatant was measured by fluorescence method (LS-55, Perkin Elmer, USA, excitation wavelength at 488 nm, and emission wavelength at 593 nm) using a calibration curve constructed from DOX standard solutions. The encapsulation efficiency (EE) and drug-loading content (DL) were calculated using the following equations:

$$\text{DL (\%)}=\frac{\text{Amount of drug in liposomes}}{\text{Amount of drug loaded liposomes}}\times 100$$

$$\text{EE (\%)}=\frac{\text{Amount of drug in liposomes}}{\text{Total amount of feeding drug}}\times 100$$

### 2.5. Redox-sensitivity of drug-loaded liposomes

The reduction-induced destabilization of Chol-SS-COS/ES-Lp was evaluated as the changes in size of the liposomes suspended in a PBS (0.1 M, pH 7.4) containing 10 mM dithiothreitol (DTT, a reducing agent). The mixture was placed in shaking water bath at 37 °C and the particle size was monitored by a Malvern Zetasizer using the dynamic light scattering (DLS) technique after 2 h and 4 h of incubation.

In parallel, 1 ml of each DOX-loaded Chol-SS-COS/ES-Lp, was placed into a dialysis bag (MWCO 10 KDa) and immersed into 30 ml of PBS (10 mM, pH 7.4) containing 0, 20 μM or 10 mM GSH at 37 °C in a shaking bed. Chol-COS/ES-Lp was used as a reference. At designed time intervals, an aliquot (1 ml) of the external buffer was removed and replaced with an equivalent volume of fresh medium. The concentrations of DOX were determined by measurement of fluorescence as described above.

### 2.6. Physical stability of DOX-loaded liposomes

The short-term physical stability of DOX-encapsulated liposomes in PBS (pH 7.4, 10 mM) containing 10% FBS stored at 4 °C was monitored by the measurement of particle size using DLS.

### 2.7. Selective *in vitro* cytotoxicity

MG63 and LO2 cells were maintained in Dulbecco’s modified Eagle medium (FBS 15%, v/v) and RPMI-1640 medium (10% FBS, v/v) respectively and both were supplemented with penicillin-streptomycin (1%, v/v) and cultured in humidified 5% CO2 at 37 °C. Medium was routinely replaced with fresh medium every 2 days. The cells in logarithmic phase of growth were cryopreserved for following research.

For cytotoxicity assay, LO2 and MG63 cells were seeded in 96-well plates at a density of 10^4^ cells/well. The cells were maintained in 5% CO_2_ at 37 °C in an automated incubator for 24 h to allow cells to attach. Following this, the cells were exposed to COS polymer, blank liposomes, free DOX or different drug-loaded liposomes which were dispersed in culture medium. After 24 h of incubation, the medium containing formulations was washed off and cell viabilities were measured using the MTT (3-(4,5-dimethylthiazol-2-yl)-2,5-diphenyltetrazolium bromide) tetrazolium reduction assay. The optical density (OD) was measured at 490 nm using a microplate reader (Spectra Max M2, Molecular Devices). All experiments were carried out six times repeatedly. Cell viability (%) of the formulation treated was assessed by comparing with the untreated cells.

### 2.8. Selective cellular uptake

To understand the mechanism of cytotoxicity the targeting effect of ES, and the reduction-responsiveness on cellular uptake of all liposomal formulations by MG63 overexpressing estrogen receptors and liver cells LO2 (bearing no estrogen receptors) was compared using confocal laser scanning microscope (CLSM) and flow cytometry (FCM).

For the FCM studies, cells were seeded in 6-well plates at 10^6^/well in the medium and cultured in 5% CO_2_ at 37 °C, overnight. Then, the cells were exposed to four different formulations dispersed in medium (free DOX, Chol-COS/DOX, Chol-SS-COS/DOX, Chol-SS-COS/ES/DOX) with a final DOX concentration of 20 μg/ml for different time points at 37 °C. Following this, the cells were washed twice with 2 ml of PBS, then detached by trypsin, and centrifuged at 1500 rpm for 5 min. Finally, the cells in pellet were re-suspended in 0.5 ml of PBS and analyzed by a FACS Calibur flow cytometer (EPICS XL, Beckman).

For CLSM analysis, cells were seeded onto clean and sterile coverslips placed in 12-well plates at an initial density of 5 × 10^5^ cells/well and cultured overnight. Cells were then treated with free DOX, or each of the four DOX containing liposomes at 37 °C for 2 h or 4 h (equivalent DOX concentration: 20 μg/ml), respectively. After incubation, the cells were washed with PBS for three times, fixed with paraformaldehyde solution (4%) for 30 min, stained by Hoechst 33342 solution (10 μg/ml) for 10 min before confocal microscopic analysis (TCS SPE, Leica, Germany).

### 2.9. Biodistribution and anti-tumor effect on MG63 tumor bearing nude mice models

Male BALB/c nude mice (18–22 g) were provided by Beijing Vital River Laboratory Animal Technology Co., Ltd, and were treated according to the protocols approved by the Experimental Animals Administrative Committee of Yantai University.

MG63 bearing mice models were established by subcutaneous inoculation of osteosarcoma MG63 (1 × 10^7^ in 100 µl McCoy’s 5 A medium) in left-hand limb of each nude mice.

#### 2.9.1. *Ex vivo* fluorescence biodistribution and tumor targeting

When tumors reached ∼300 mm^3^, mice were intravenously injected by the free DiR and DiR-loaded liposomes at the DiR dose of 500 μg/ml, 0.2 ml. The near-infrared (near-IR) optical images were taken on Carestream Molecular Imaging FX PRO (Carestream Health, Inc., NY). At 4-h postinjection, the mice were euthanized, and tumors as well as other tissues were harvested for *ex vivo* imaging.

#### 2.9.2. *In vivo* anti-tumor efficacy

The MG63 tumor bearing mice (with tumor volumes reached 100 mm^3^ approximately) were randomly assigned to the following five treatment groups (*n* = 5). Animals were treated with saline, free DOX and three different liposomal formulations at a dose of DOX of 5 mg/kg which were administrated via the tail veins every 4 day for four times. The tumor size (length and width) was closely monitored every 2 days and tumor volume was calculated as 0.5 × length × width^2^. Tumor volume on Day 0 was normalized to 100% for all groups. On the 24th day of treatment, animals were sacrificed, and the tumors were excised, weighed and photographed.

### 2.10. Statistical analysis

All studies were carried out in triplicate. One-way analysis of variance (ANOVA) was used to determine the difference of groups, and data were considered to be significant at *p* < .05.

## 3. Results and discussions

### 3.1. Structural characterization of DSPE-PEG_2000_-ES

**Figure 1.**
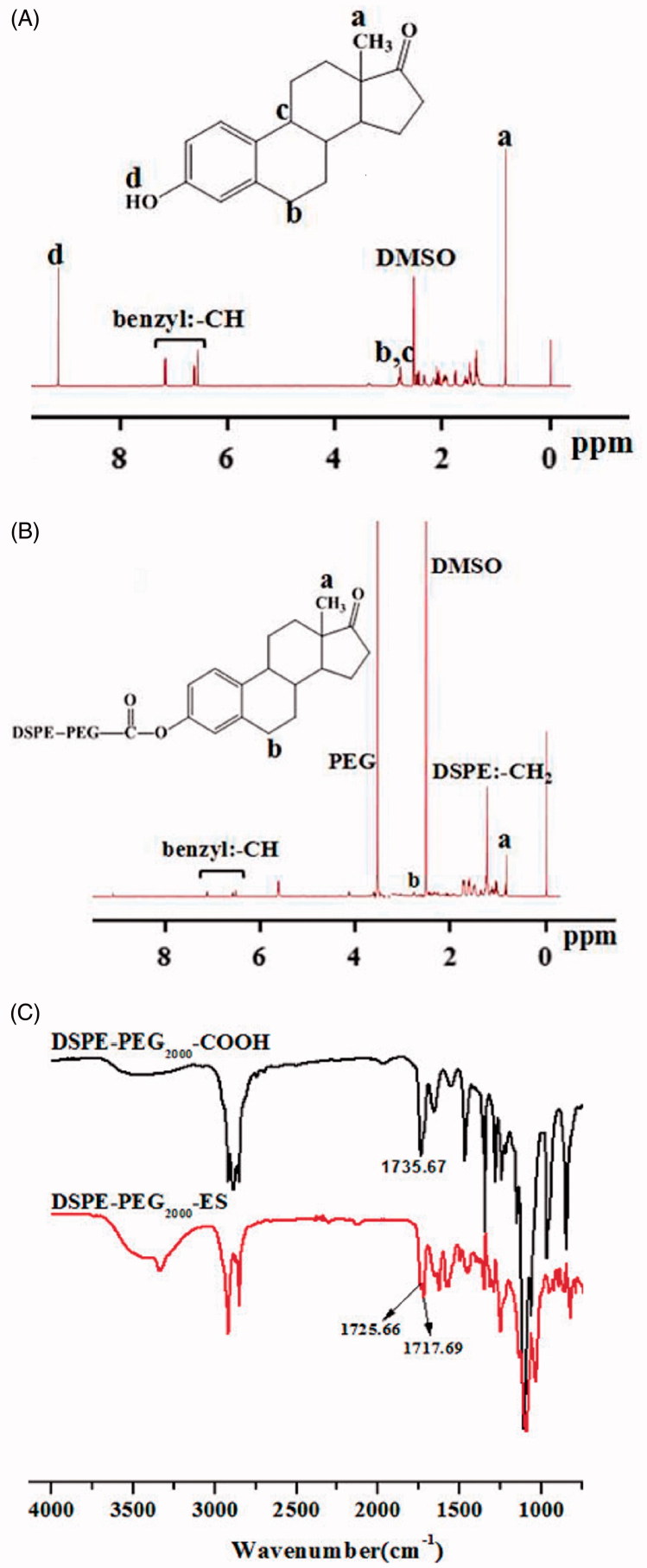
^1^H NMR spectra of estrone (A) and DSPE-PEG_2000_-ES (B), and FT-IR spectra of DSPE-PEG_2000_-COOH and DSPE-PEG_2000_-ES (C).

DSPE-PEG_2000_-ES was successfully synthesized by a single-step reaction, as confirmed with the ^1 ^H NMR spectra (Figure 1(A,b)), in which the peaks at 9.02 ppm of estrone disappeared, and the methylene peak of mPEG_2000_ was shown at 3.53. In addition, the peaks at 7.06 ppm, 6.52 ppm, 6.64 ppm of estrone (benzene ring) were found, indicating the successful introduction of targeting molecule to DSPE-PEG_2000_.

In addition, in the FT-IR (Figure 1(C)), DSPE-PEG_2000_-COOH shows IR peaks at 1735.67 cm^−1^ corresponding to the carboxylic group (-COOH). After the reaction with ES, this peak disappeared, and a new peak at 1725.66 cm^−1^ (ester bond) was shown. The peak at 1717.69 cm^−1^ represents the ketonic group of estrone, suggesting the formation of DSPE-PEG_2000_-ES.

### 3.2. Physicochemical characterization of liposomes

Liposomes were successfully prepared used for ethanol injection method. The liposomes without COS or PEG-ES had a ζ potential of −6.52 ± 1.86 mV and a size of 74.2 ± 2.2 nm. The modified liposomes showed positive ζ potential and increased particle size but still around 110 nm with a relative narrow size distribution (PDI < 0.2) (Table 1). These results suggested that COS was successfully tethered on the surface of cholesterol. In addition, DOX was loaded into the liposomes using the ammonium sulfate gradient method with large EE (>85%) and DL (12.9–15%, w/w).

**Table 1.** The size, polydispersity index (PDI), zeta potential, drug-loading content (DL) and encapsulation efficiency (EE) of different liposomes.

Samples	Particle size (nm)	PDI	Zeta potential (mV)	DL (% w/w)	EE (% w/w)
Chol-SS-COS-Lp	105.7 ± 0.42	0.197 ± 0.008	33.9 ± 0.737	12.9 ± 2.34	85.4 ± 1.36
Chol-COS/ES-Lp	104.8 ± 1.35	0.188 ± 0.009	35.9 ± 2.17	14.7 ± 0.02	89.9 ± 0.17
Chol-SS-COS/ES-Lp	110.0 ± 1.5	0.177 ± 0.002	36.9 ± 1.61	15.1 ± 0.84	100.2 ± 2.71

The data were represented as the mean ± standard deviation (*n* = 3).

**Figure 2.**
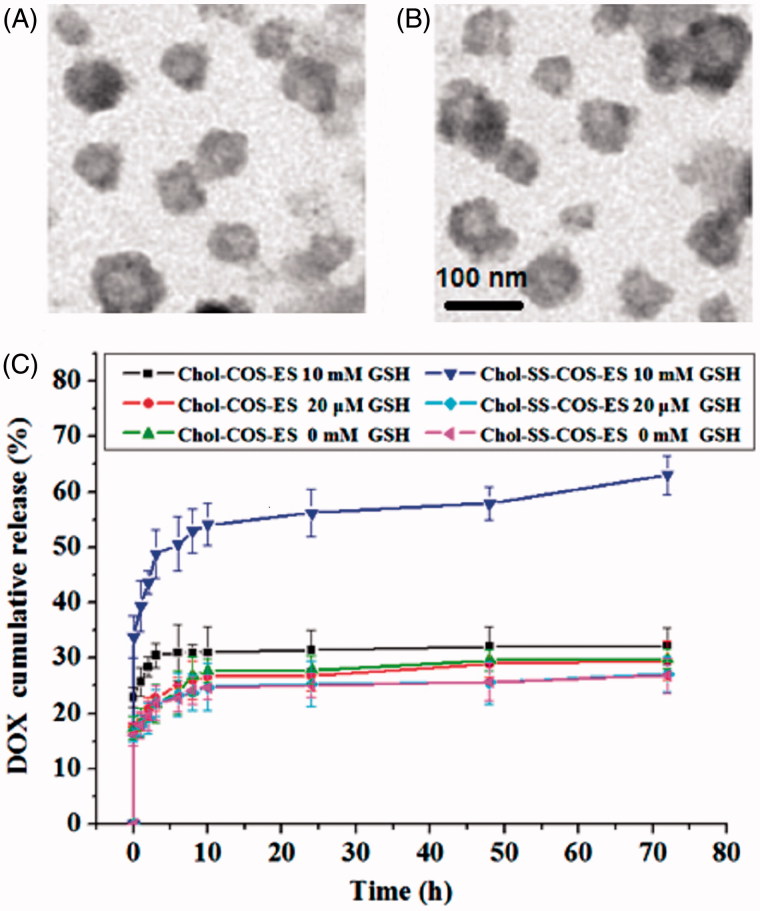
TEM images of Chol-COS/ES-Lp (A) and Chol-SS-COS/ES-Lp (B), and their drug release profiles in PBS (10 mM, pH 7.4) containing GSH at various concentrations (0, 20 μM or 10 mM) (C).

The TEM images (Figure 2) confirmed the formation of spherical liposomes. The particle size was slightly smaller than that observed by DLS, possibly due to shrinkage of the coating polymers in a dry state.

The liposomes suspended in PBS (pH 7.4, 10 mM) containing 10% FBS to mimic *in vivo* environment showed good stability with no significant changes in the average particle size or drug leakage over 15 days.

### 3.3. Reduction-sensitivity of DOX-loaded liposomes

To evaluate the redox-sensitive drug release behavior, the liposomes were placed at 10 mM DTT in PBS (10 mM, pH 7.4), and the size change was measured by DLS. At 10 mM DTT, Chol-SS-COS/ES-Lp aggregated rapidly, and the size of liposomes increased from 119.3 ± 1.4 nm to 125.3 ± 1.54 nm at 2 h and 228.1 ± 4.95 nm after 4 h. These results confirmed that Chol-SS-COS/ES-Lp were dissociated in the presence of DTT, most possibly due to the cleavage of the -SS- bonds.

### 3.4. *In vitro* GSH-responsive drug release

The release behavior of DOX-loaded liposomes in presence of GSH (pH 7.4) exhibited time-dependence and reduction-sensitivity (Figure 2(C)). Obviously, different release behaviors were observed in different concentration of GSH due to the breakage of disulfide bond. In all cases, all tested formulations possess sustained release profiles, and in 10 mM GSH, 58% of DOX was released from Chol-SS-COS/ES-Lp over 10 h. At 72 h, 65% DOX were released from the Chol-SS-COS/ES-Lp. In contrast, in the absence or low convention of GSH (20 μM), the accumulative release of DOX was lower than 40% at 72 h. In contrast, the control formulation, Chol-COS/ES-Lp, had drug release lower than 30% in all cases.

### 3.5. Selective *in vitro* cytotoxicity

In the MTT assay, all formulations showed DOX dose-dependent toxicity manner to both LO2 and MG63 cells with the concentration ranging from 0.001 to 10 μg/ml. The order of cytotoxicity was ranked as: free DOX > Chol-SS-COS/ES/DOX > Chol-SS-COS/DOX > Chol-COS/DOX. In addition, Chol-SS-COS/ES/DOX liposomes had favorably higher cytotoxicity against cancerous MG63 cells than to LO2 liver cells under the same condition. The half inhibitory concentrations (IC50) of Chol-SS-COS/ES/DOX-Lp was 0.89 ± 0.25 μg, much lower than that of Chol-SS-COS/DOX-Lp (1.70 ± 0.24 μg/ml) and Chol-COS/DOX and (2.49 ± 0.17 μg/ml) on MG63 cells. The corresponding values to LO2 cells were higher with no differences between the liposome formulations (2.87 ± 0.15 μg/ml vs. 2.76 ± 0.12 μg/ml). Under the same condition, the IC50 values of free DOX was 0.61 ± 0.04 μg/ml and 1.15 ± 0.14 μg/ml to MG63 cells LO2 cells, respectively (Yin et al., 2017).

Additionally, the cell viability remained above 85% following treatment with blank liposomes or free COS for 48-h incubation, demonstrating COS possess good biocompatibility.

### 3.6. *In vitro* cellular uptake

**Figure 3.**
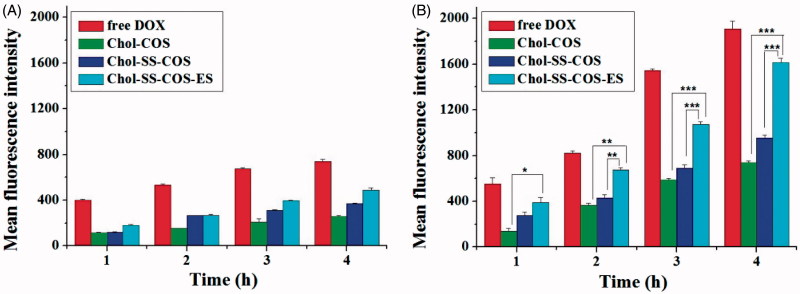
Flow cytometry analysis of LO2 (A) and MG63 cells (B) incubated with free DOX and three different formulations for 1, 2, 3 and 4 h. DOX dosage was 20 μg/ml. Data represent the mean ± SD (*n* = 3). *p < .05; **p < .01 and ***p < .001.

Flow cytometry (Figure 3) showed that the cellular uptake of DOX by both cell types possessed a time-dependent manner, and the cellular uptake rates were ranked in the order: free DOX > Chol-SS-COS/ES-Lp > Chol-SS-COS-Lp > Chol-COS-Lp for both cells. Moreover, the mean fluorescence intensity of DOX in MG63 cells was significantly higher than that in LO2 cells treated with all the liposomes, in particular, Chol-SS-COS/ES-Lp, suggesting their selectivity for tumor cells, attributed to the function of estrone and the reduction sensitivity.

**Figure 4.**
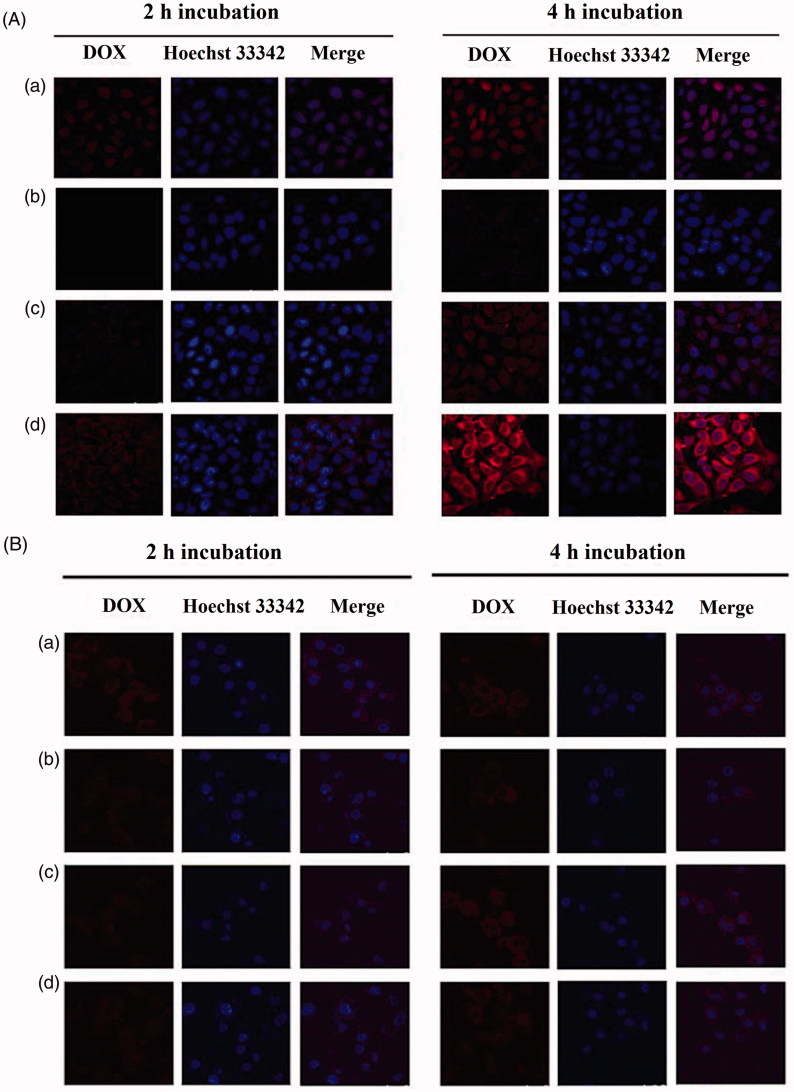
Confocal microscopy images of (A) MG63 cells and (B) LO2 cells. Cells were treated with free DOX solution (a), Chol-COS/DOX-Lp (b), Chol-SS-COS/DOX-Lp (c) and Chol-SS- COS/ES/DOX-Lp (d) for 2 h and 4 h, respectively.

The cellular uptake of different formulations by MG63 and LO2 cells was further investigated with confocal laser scanning microscopy by visualizing the DOX fluorescence intensity (Figure 4). Similar to the flow cytometry results, the fluorescence intensity of DOX was enhanced in both of MG63 over time, for all DOX formulations. Interestingly, DOX was mainly observed to be distributed in cellular nuclei of MG63 cells following the treatment with free DOX and mainly distributed in cytoplasm of MG63 following the treatment with liposomes. In addition, the order of the fluorescence intensity of DOX is ranked Chol-SS-COS/ES-Lp > DOX solution > Chol-SS-COS-Lp > Chol-COS-Lp. The stronger fluorescence intensity observed in MG63 cells than in LO2 cells following treatment with Chol-SS-COS/DOX and Chol-SS-COS/ES/DOX, provided evidence for the over expression of estrogen receptors on the surface of MG63 cells (Cao et al., 2003), rather than LO2 cells.

### 3.7. Biodistribution and anti-tumor efficacy in MG63 tumor bearing mice

**Figure 5.**
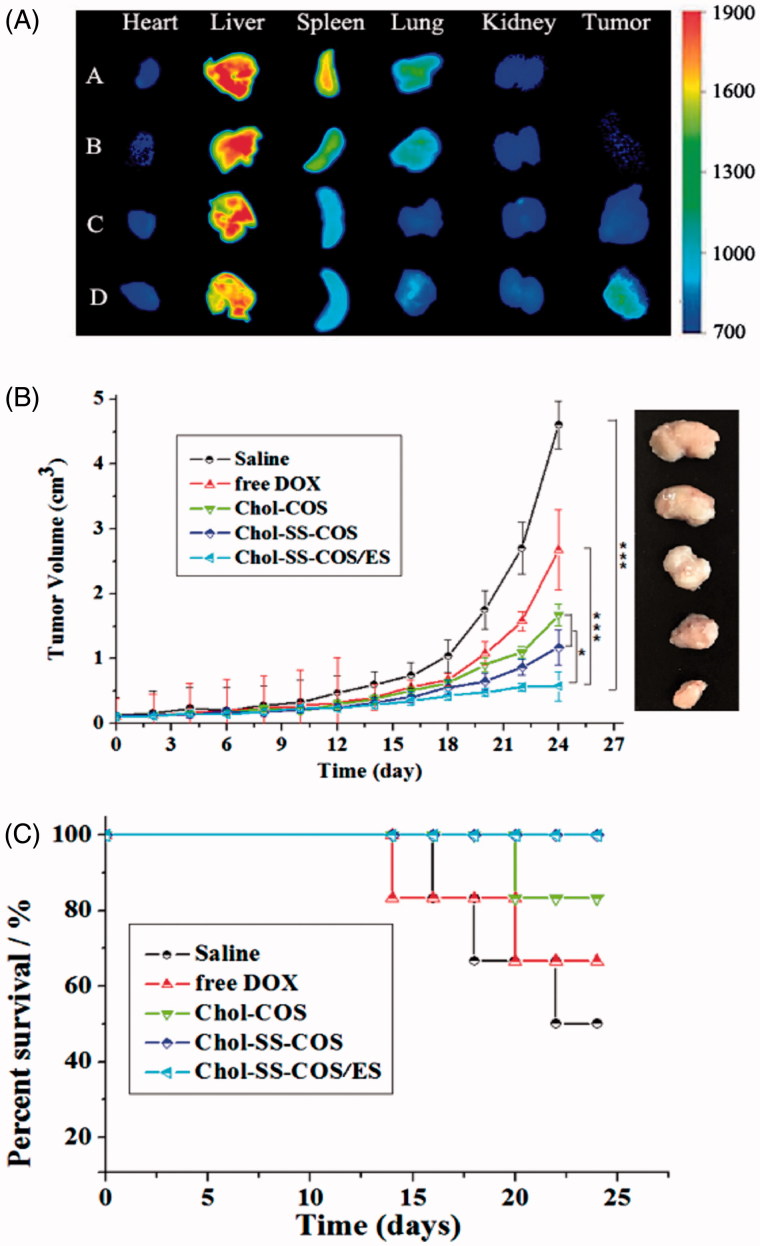
(A): *Ex vivo* imaging of tumors and major organs of MG63 tumor-bearing nude mice at 4-h postinjection of A: free DiR, B: Chol-COS/DiR-Lp. C: Chol-SS-COS/DiR-Lp; D: Chol-SS-COS/ES/DiR-Lp at DiR dose of 500 μg/ml, 0.2 ml. (B) The antitumor effects of different DOX formulations at in MG63 tumor bearing nude mice (mean ± SD, *n* = 5 initially); and (C) Animal survival rates. *p < .05, **p < .01, ***p < .001. The drug dosing at 5 mg/kg of DOX per injection started on Day 0 and repeated on Days 4, 8 and 12.

As shown in Figure 5(A), fluorescent dye DiR was mainly distributed in liver of to MG63 tumor-bearing nude mice at 4-h postinjection of free DiR. The result of DiR-loaded Chol-COS-Lp and Chol-SS-COS-Lp group exhibited that DiR also largely accumulated into the liver, and while weaker fluorescent intensity was observed in tumor. In contrast, strongest fluorescence intensity was observed in tumors treated with Chol-SS-COS/ES-Lp while little observed in heart, spleen and kidney.

*In vivo* anticancer activity was carried out in MG63 tumor-bearing nude mice via tail vein injection of different formulations every 4 days for four times. As shown in Figure 5(B), the tumor size of saline group increased significantly over time. In comparison, the liposomal formulations exhibited more potent antitumor activities with Chol-SS-COS/ES-Lp being most potent. At the end of the experiment, the tumor weight (g) were measured as 2.84 ± 0.23, 1.90 ± 0.16, 1.38 ± 0.13, 1.12 ± 0.02 and 0.70 ± 0.12 for the groups treated with normal saline, free DOX, Chol-COS-Lp, Chol-SS-COS-Lp and Chol-SS-COS/ES-Lp, respectively. In addition, along with other GSH-responsive liposomes Chol-SS-COS-Lp, Chol-SS-COS/ES-Lp treatment resulted in 100% animal survival rate in contrast to free DOX and Chol-COS groups (Figure 5(C)).

## 4. Discussion

In this paper, we chose a partially hydrolyzed product of chitosan, COS with MW 2–5 KDa as alternative to PEG to coat liposomes via disulfide bond (-SS-) and equipped the nanocarriers with a ligand (estrogen) (Chol-SS-COS/ES-Lp) to target osteosarcoma. Estrone was successfully conjugated with DSPE-PEG_2000_ (Figure 1) which was incorporated in liposomes and was possibly protected in the COS layers, whereas the COS was grafted on the surface via the reaction of –NH_2_ groups with Chol-SS-COOH in the liposomes (Yin et al., 2017). The stable Chol-SS-COS/ES/DOX liposomes with a high drug loading of DOX had a size around 110 nm and therefore can exploit the EPR effect. The estrone and -SS- bonds were to impart tumor-targeting effect as well as redox-sensitive properties respectively to the liposomal system. As only 3% mol of PEG2000 (usually 5%) was used with estrone conjugated to the end, and being sandwiched in the COS polymers such design may avoid the ‘PEG dilemma’, at least to some extent.

The addition of DSPE-PEG-ES did not change the cationic property (zeta potential +36 mV, Table 1). This would be beneficial for a preferential uptake in angiogenic tumor vessels (Abu Lila et al., 2010) as well as the negatively charged cancer cells (Chen et al., 2016). As the *in vitro* release profile shown good stability of vesicles with <30% drug released within 10 h in PBS (10 mM, pH 7.4) but released 50% of DOX within 4 h in the presence of 10 mM GSH (Figure 2(C)). Therefore, such liposomes are assumed to destabilize in the reduction-sensitivity environment of cancer cells, rapidly releasing their contents due to rapid cleavage of -SS- bonds (Jhaveri et al., 2014).

There was remarkable increase in cellular uptake of Chol-SS-COS/ES compared with Chol-SS-COS in the MG63 cells (Figures 3 and 4), demonstrated that Chol-SS-COS/ES liposomes served as an efficient vehicle to transport the payload into cells and release to the cytosol. The Chol-SS-COS/ES liposomes were internalized via receptor-mediated endocytosis, subsequently, in the GSH environment of cytoplasm triggered the release of DOX due to the cleavage of –SS-bond thus detachment of COS. In contrast, the cellular uptake by LO2, normal liver cells, was much weaker for all formulations and estrone or GSH-sensitivity did not show any effects. This helped explain the cytotoxicity data which showed that Chol-SS-COS/ES/DOX were most toxic to MG63 cells and less toxic to LO2 cells, as shown in the IC_50_ values. The study confirmed that the cell growth inhibition was owing to the cellular internalization of Chol-SS-COS/ES/DOX via estrogen receptor-mediated targeting and the cleavage of disulfide bonds responding to GSH-sensitive condition. The results also showed the empty liposomes were nontoxic to normal cells.

It is worth of mentioning that Chol-SS-COS/ES produced strongest fluorescence intensity in the MG63 cells than the free DOX following exposure to cells for up to 4 h (Figure 4). As only the non-ionized form of DOX (basic pK_a_ of 8.3) distributes on both sides of the cell membrane, ionized DOX is sequestered in the extracellular environment (in tumor pH could be 6.5) or organelles with low pH such as endosomal and lysosomes (‘Ion Trapping’ phenomenon) (Wojtkowiak et al., 2011). This may result in drug resistance. Therefore, intracellularly delivery with liposomes can be efficient to overcome the ‘Ion Trapping’ phenomenon, if DL is sufficient (Xu et al., 2014). These results confirmed the role of estrogen-receptor-mediated endocytosis in efficient intracellular delivery of the DOX via estrogen receptor-targeted liposomes. In addition, the formulations showed larger discrepancy in cellular uptake than in the cytotoxicity study. This is more likely due to the fact the *in vitro* cytotoxicity study was carried out following longer time of drug exposure (24 h) to the cells in which case all liposomes.

Following if injection to MG63 tumor-bearing nude mice (Figure 5(C)), strong fluorescence intensity was observed in tumors treated with Chol-SS-COS/ES-Lp while little observed in heart, spleen and kidney, predicted a high efficacy and low side effects.

The *in vivo* efficacy of DOX-loaded Chol-SS-COS/ES-Lp was further compared with Chol-SS-COS-Lp and Chol-COS-Lp on MG63 osteosarcoma model (Figure 5(B)). Only slight inhibition of tumor growth was observed after injection of free DOX and Chol-COS-Lp. Notably, Chol-SS-COS/ES-Lp showed great inhibition of tumor growth along with 100% survival rate (Figure 5(C)). The high antitumor activity of the Chol-SS-COS/ES-Lp can be attributed to a higher accumulation in cancer cells via receptor-mediated uptake and accelerated release of DOX after endocytosis. The high survival rate of the Chol-SS-COS/ES-Lp treated animals again verified the safety profile of COS as an alternative of PEG.

Overall, this study demonstrated the COS-based estrogen-functionalized redox-sensitive nano-liposomes as potential drug delivery system for improved antitumor outcomes. However, data should be carefully interpreted as there are are number of major ‘pitfalls’ in clinical translation, for example, the current animal models cannot fully reflect human cancer pathology and may overly present the EPR effect (Lammers et al., 2016; Mitragotri et al., 2017).

## 5. Conclusions

A reduction-responsive and estrogen functionalized liposomes drug delivery system grafted with biocompatible aninoplysaccharide polymer (COS) was developed and was demonstrated for its potential for intracellular drug delivery to osteosarcoma MG63 cells. The Chol-SS-COS/ES/DOX liposomes with a high drug loading of DOX had a size around 110 nm and therefore can exploit the EPR effect, as demonstrated in the mouse xenograft models. The multifunctional liposomes had rapid cellular uptake, mediated by estrogen-receptors followed by rapid DOX release in response to 10 mM GSH due to the cleavage of disulfide bonds which allowed COS detached from liposomes within cells, thus exerted higher cytotoxicity to MG63 cells than LO2 liver cells *in vitro* and *in vivo*. The COS-modified liposomes also demonstrated great safety profile in mice. In summary, the significant inhibitory effects on tumor growth of the redox-responsive and estrogen receptor-targeted cationic liposomes showed great potential for delivery drug to estrogen receptor-expressing tumors such as osteosarcoma.
